# Kinome multigenic panel identified novel druggable EPHB4‐V871I somatic variant in high‐risk neuroblastoma

**DOI:** 10.1111/jcmm.15297

**Published:** 2020-04-26

**Authors:** Immacolata Andolfo, Vito A. Lasorsa, Francesco Manna, Barbara E. Rosato, Daniela Formicola, Achille Iolascon, Mario Capasso

**Affiliations:** ^1^ Department of Molecular Medicine and Medical Biotechnologies University of Naples Federico II Naples Italy; ^2^ CEINGE Biotecnologie Avanzate Naples Italy; ^3^ IRCCS SDN Naples Italy

**Keywords:** drug, EPHB4, high‐risk neuroblastoma, kinases, personalized medicine, somatic mutation

## Abstract

Neuroblastoma (NB) is the most common extracranial neoplasm in children. The overall outcome for high‐risk NB patients is still unacceptable, therefore, it is critical to deeply understand molecular mechanisms associated with NB, which in turn can be utilized for developing drugs towards the treatment of NB. Protein kinases (TKs) play an essential role in the regulation of cell survival and proliferation. Different kinases, such as anaplastic lymphoma kinase (ALK), Aurora kinase, RET receptor tyrosine kinase, are potential therapeutic targets in various cancers, including NB. We analysed a cohort of 45 high‐risk NB patients and 9 NB cell lines by a targeted—(t)NGS custom gene panel (genes codifying for the kinase domains of 90 TKs). We identified somatic variants in four TK genes (*ALK, EPHB4, LMTK3* and *EPHB6*) in NB patients and we functionally characterized an interesting somatic variant, V871I, in *EPHB4* gene. EPHB4 plays a crucial role in cardiovascular development and regulates vascularization in cancer‐promoting angiogenesis, tumour growth and metastasis. Several EPHB4 mutations have previously been identified in solid and haematological tumour specimens but EPHB4 mutations were not described until now in NB. Interestingly, a re‐analysis of public CGH‐array showed that the *EPHB4* gain is associated with advanced diseases in NB. We further demonstrated that higher *EPHB4* expression is correlated to stage 4 of NB and with poor overall survival. Additionally, we also revealed that the EPHB4‐V871I accounts for increased proliferation, migration and invasion properties in two NB cell lines by acting on *VEGF, c‐RAF* and *CDK4* target genes and by increasing the phosphorylation of ERK1‐2 pathway. The use of two EPHB4 inhibitors, JI‐101 and NVP‐BHG712, was able to rescue the phenotype driven by the variant. Our study suggested that EPHB4 is a promising therapeutic target in high‐risk NB.

## 1 INTRODUCTION

Neuroblastoma (NB) is the most common extracranial neoplasm in children and contributes to about 15% of all paediatric cancer‐related deaths.^1^ Despite major advances in therapies over the past decade, the overall outcome for high‐risk NB patients is still unacceptable.^2^, ^3^, ^4^, ^5^ Current therapies include chemotherapy drugs that are highly toxic for healthy cells and have significant long‐term side effects.^6^ Therefore, developing novel targeted therapies for high‐risk NB is critical to achieve higher efficacy and to alleviate adverse effects. Future improvements in high‐risk NB outcomes will require the identification of disease and patient‐specific oncogenic variations that can be druggable.^7^ Among high‐risk patients, gene signatures can identify children with high‐risk disease who would benefit from new and more aggressive therapeutic approaches.^2^ *MYCN* amplification is a strong characteristic of high‐risk NB patients and is a genetic marker of disease.^3^ However, finding therapeutic strategies to directly target *MYCN* is a difficult task due to its protein structure. High‐throughput sequencing‐based studies have highlighted that recurrent mutations of single genes are infrequent in primary NB with activating mutations in *ALK,* inactivating mutations in *ATRX* and *TERT* rearrangements being the most frequent.^5^, ^8^, ^9^, ^10^ RAS/P53 and FA and RAC pathways are among the most significantly mutated pathways in NB.^11^, ^12^, ^13^, ^14^, ^15^

Kinases play a crucial role in the regulation of cell survival and proliferation.^16^ Different kinases, such as anaplastic lymphoma kinase (ALK),^13^, ^17^ Aurora kinase,^14^ RET receptor tyrosine kinase,^15^ are potential therapeutic targets in various cancers, including NB.^18^, ^19^, ^20^ Indeed, molecules as ALK inhibitors were found to be appropriate in patients whose tumours harbour activating ALK mutations. Although some mutations seem to be resistant to current ALK inhibitors (ie F1174L), new drugs have already been formulated to overcome this resistance.^17^ Moreover, these drugs are actively being evaluated in the New Approaches to Neuroblastoma Therapy (NANT) consortium.^19^

In this study, we analysed a cohort of 45 high‐risk NB patients and 9 NB cell lines by targeted (t)‐NGS customized TK domains panel. We identified a somatic variant p.V871I in *EPHB4* gene. EPHB4 plays a crucial role in cardiovascular development and regulates vascularization in cancer‐promoting angiogenesis, tumour growth and metastasis.^21^ Several EPHB4 mutations have previously been identified in solid and haematological tumour specimens.^21^, ^22^, ^23^ Many other EPHB4 variants have been identified in other types of tumours and cell lines and catalogued in “The Cancer Genome Atlas project.”^24^ However, EPHB4 mutations were not described until now in NB. We demonstrated that higher *EPHB4* expression is correlated with poor overall survival. Moreover, the functional study highlighted the role of the variant by increasing proliferation, migration and invasion in NB cells. Of note, the treatment of the cells with two EPHB4 inhibitors, JI‐101 and NVP‐BHG712, was able to rescue the phenotype driven by the variant suggesting that EPHB4 is a promising therapeutic target in high‐risk NB.

## 2 MATERIALS AND METHODS

### 2.1 Targeted—(t)NGS panel design

The “kinome” custom sequencing panel was designed to cover the kinase domains of TKs. KinBase (http://kinase.com/; a database of protein kinases) was queried to retrieve the coding regions of kinase domains.^20^ Six hundred seventeen coding regions with a mean length of 260.66 bp (40‐9408 bp) were selected, and their genomic coordinates were extended of 50 bp up‐ and down‐stream to get a final target of about 222.5 Kb.

### 2.2 High throughput sequencing

For high‐risk NB samples, kinome targeted regions were captured and enriched using the SeqCap EZ Library SR (Roche NimbleGen). Captured DNAs were subjected to massively parallel sequencing using an Illumina HiSeq 1000 obtaining 90 bp paired‐end reads.

For NB cell lines, kinome targeted regions were captured and enriched using the Agilent HaloPlex target enrichment system (Agilent Technologies) according to the manufacturers’ protocol. The sequencing was performed on an Illumina HiSeq 1000 yielding 90 bp paired‐end reads.

### 2.3 Sequencing data processing and mutation calling

Illumina paired‐end reads of NB samples were mapped versus the reference genome (GRCh37/hg19 downloaded from UCSC Genome Browser) using the BWA (Burrows‐Wheeler Aligner)^25^ algorithm with default parameters. Alignment information, stored in BAM (Binary Alignment‐Map) files, for tumour and control tissue pairs, was piled up with SAMTools,^26^ and variants were called using VarScan2 (“somatic” algorithm)^27^ with default parameters.

Sequencing reads of NB cell lines were processed with the Agilent SureCall software (v1.1), which implements BWA for the mapping step and SAMTools for the variant calling.

The quality check of sequences was performed with FASTQC software whereas BAM files were checked with BamTools, SAMTools and GATK.

The raw genetic variants identified and stored in VCF (Variant Call Format) files were annotated using ANNOVAR.^28^ Annotated variants were strongly filtered to get a high confidence set of somatic changes. We kept only the variants: (a) with variant call *P*‐value ≤ .01 (Fisher Exact Test implemented in VarScan2 for somatic variant calling and chi‐square test of variant call quality implemented in SureCall software); (b) not registered in dbSNP135; (c) rare, with allele frequency lower than 0.01 in human population variant databases (1000 Genome Project, ESP 6500, ExAC, CG46); (d) without sequencing strand bias (variants with a strand bias over the 90% were set aside to reduce false‐positive calls); (e) protein sequence impacting (eg missense); (f) with CADD score greater than 10 because these variants were considered as pathogenic. The set of variants obtained was manually curated and visually inspected with the IGV—Integrative Genomics Viewer.^29^

### 2.4 Public data sets analysis

Gene expression, survival (GEO Id: GSE45547; n = 649) and copy number analysis (GEO Id: GSE103123; n = 553) were performed by using the R2 Genomics Analysis and Visualization Platform (http://r2.amc.nl). The overall and the event‐free (relapse‐free) survival probability were calculated by using the Kaplan‐Meier method, and the significance of the difference between Kaplan‐Meier curves was calculated by the log‐rank test. We defined “high” and “low” groups containing samples with *EPHB4* expression greater than, or lower than, its median value, respectively. Copy number gains for GSE103123 (a data set including 346 aCGH and 207 SNP arrays) were defined as in Depuydt et al^30^ The selected DNA region, downloaded from R2 web tool, ranged from 100350145 to 100960971 on chromosome 7q. The cut‐off to call copy number gains was set at Log Ratio = 0.2 and 0.15 for aCGHs and SNP arrays, respectively.

In gene expression and copy number analysis, we assessed the significance of differences between groups by t test and chi‐square test, respectively. Statistical significance was set at 5%.

### 2.5 Cell culture

SKNBE2 and SHSY5Y human NB cell lines were obtained from the cell bank of Ceinge‐Biotecnologie Avanzate. SKNBE2 cells were maintained in Dulbecco's modified Eagle's medium (DMEM; Sigma), and F12 Medium (Sigma) supplemented with 10% foetal bovine serum (FBS), 10 U/mL penicillin and 0.1 mg/mL streptomycin (Sigma). SHSY5Y cells were maintained in Dulbecco's modified Eagle's medium (DMEM; Sigma) supplemented with 10% FBS, 10 U/mL penicillin and 0.1 mg/mL streptomycin.

### 2.6 Patients

A total of 45 surgical NB specimens were used for the kinome panel. All the specimens were obtained at the time of diagnosis, before radiation therapy or chemotherapy, and were subjected to histopathological review according to the WHO criteria. DNA was obtained for genetic analysis from patients after signed informed consent, according to the Declaration of Helsinki, and as approved by local university ethical committees.

### 2.7 RNA isolation, cDNA preparation and quantitative real‐time PCR

Analysis total RNA was extracted from the cell lines using Trizol reagent (Invitrogen). Synthesis of cDNA from total RNA (2 mg) used SuperScript II First‐Strand kits (Invitrogen). Quantitative real‐time PCR (qRT‐PCR) was performed using the SYBR‐green method, following standard protocols with an Applied Biosystems ABI PRISM 7900HT Sequence Detection system. Relative gene expression was calculated using the 2^−ΔCt^ method, where ΔCt indicates the differences in the mean cycle threshold (Ct) between selected genes and the internal control.^31^ QRT‐PCR primers for each gene were designed using Primer Express software, version 2.0 (Applied Biosystems). Primer sequences are available upon request. The significance of the gene expression differences was determined using Student's *t* tests; statistical significance was established at *P* < .05.

### 2.8 Vector cloning and site direct mutagenesis

cDNA encoding full‐length wild‐type *EPHB4* (6584384) was obtained by Origene and then subcloned into pCMVtag1 vector (Agilent) using the HindIII‐SalI restriction sites. The point mutation c.G2611A, p.V871I, was introduced into pCMVtag1‐EPHB4 by site‐directed mutagenesis, as previously described.^32^

### 2.9 Western blotting

Total lysates of 50 µg were loaded and run on 12% polyacrylamide gels, which were then blotted onto polyvinylidene difluoride membranes (BioRad). These membranes were incubated with the following antibodies: polyclonal rabbit anti‐EPHB4 (1:500; 20883‐1‐AP, Proteintech); rabbit polyclonal anti‐pErk1/pErk2 (Cat. No ab32538; 1:250 dilution; AbCam) and rabbit polyclonal anti‐Erk1/2 (Cat. No ab17942; 1:1000 dilution; AbCam). An anti‐beta‐actin antibody (1:5000; Sigma) was used in control for the equal loading of the total lysates.

### 2.10 Cell proliferation assay

The proliferation of SKNBE2 and SHSY5Y cells was assessed by seeding the cells stably expressing empty vector (EV), EPHB4‐WT and EPHB4‐V871I in medium containing 10% FBS in 96‐well plates (20 000 cells/well). Cell viability was analysed after 72 hours using the MTS [3‐(4,5‐dimethylthiazol‐2‐yl)‐5‐(3‐carboxymethoxyphenyl)‐2‐(4‐sulfophenyl)‐2‐H‐tetrazolium] assay with the Cell Titre 96 Aqueous One Solution Cell Proliferation Assay (Promega), and cell survival curves were established as previously described.^33^

### 2.11 Colony formation in soft agar

SKNBE2 and SHSY5Y cells stably expressing EV, EPHB4‐WT and EPHB4‐V871I were plated at 2 × 10^5^ cells/well in triplicates in 0.35% agarose‐coated 6‐well plates in the presence of medium containing 10% foetal bovine serum. After 2 weeks, the colonies were stained with crystal violet, and the numbers of colonies were counted.

### 2.12 Monolayer wound‐healing assay

SKNBE2 and SHSY5Y cells stably expressing EV, EPHB4‐WT and EPHB4‐V871I were plated in wells of a 6‐well culture dish. Three parallel scratch wounds of approximately 400 mm width were made perpendicular to the marker lines with a P200 pipette tip (Corning). The wounds were observed after 48 hours using phase‐contrast microscopy as previously described.^34^

### 2.13 Migration assays

Migration of SKNBE2 and SHSY5Y cells stably expressing EV, EPHB4‐WT and EPHB4‐V871I through membranes with 8 µm pores was assessed using Transwell filter inserts assembled in 24‐well plates (Corning). Cells (7 × 10^5^ in 200 mL serum‐free medium) were then placed into the upper well of the membrane. Medium (500 mL) containing 10% FBS was added to the lower chamber as the chemoattractant. The assay plates were incubated at 37°C and 5% CO_2_ for 16 hours for SHSY5Y cells and for 4 hours for SKNBE2 cells. Quantification of migration through the porous membranes was carried out by counting the stained cells (0.1% crystal violet/20% methanol) using a microgrid as previously described.^34^

### 2.14 Availability of data and materials

The NGS data sets generated and analysed during this study are available from the corresponding author on reasonable request. Public data and data repositories are referenced within the manuscript.

### 2.15 Statistical analysis

All the data are presented as means + standard errors. Statistical significance was calculated using Student's t test. *P*‐value < .05 was considered statistically significant.

## 3 RESULTS

### 3.1 Kinome sequencing and identification of EPHB4‐V871I mutation in NB patients

We performed targeted sequencing of TK domains on a total of 45 NB normal‐primary tumour matched pairs and 9 NB cell lines. All tumour samples were high‐risk patients according to the COG Risk Group Classification System (Table S1). Our NGS panel comprised the kinase domains of all 90 members of the TK gene superfamily (Table S2). After the sequence alignment, the mean read depth was about 360x and 1213x for NB samples and NB cell lines, respectively. The multigene panel showed high sensitivity and specificity. Indeed, on average, the 97.08% and the 98.61% of analysable target bases were covered by at least 20 reads in NB samples and NB cell lines, respectively (Tables S3 and Table S4). The somatic variant calling returned a total of 796 (17.69 per sample) exonic variants. After stringent filtering steps (see Material and Methods), we obtained five somatic mutations in four TK genes (Table 1). We found two missense mutations in *ALK*, one of these (F1174L) is the most frequent in NB.

**TABLE 1:** Kinome somatic mutations found in NB samples and cell lines

Sample, Cell Line	Gene	Genomic change	cDNA change	Protein change	Exon	CADD	Max Population Frequency	Cosmic	*P*‐value
sample_1	*EPHB4*	chr7:100403190:C>T	c.G2611A	p.V871I	15	29.6	—	—	4.05 × 10^−311^
sample_2	*LMTK3*	chr19:49002846:C>G	c.G1567C	p.A523P	12	15.49	—	—	3.04 × 10^−04^
sample_5	*ALK*	chr2:29436860:A>C	c.T3733G	p.F1245V	24	32	—	COSM28499	1.1 × 10^−15^
sample_32	*ALK*	chr2:29443695:G>T	c.C3522A	p.F1174L	23	25.3	—	COSM28055	2.5 × 10^−21^
sample_9	*EphB6*	chr7:142566336:G>T	c.G1249T	p.A417S	11	11.51	—	—	7.89 × 10^−31^
KELLY	*ALK*	chr2:29443695:G>T	c.C3522A	p.F1174L	23	25.3	—	COSM28055	—
LAN1	*ALK*	chr2:29443695:G>T	c.C3522A	p.F1174L	23	25.3	—	COSM28055	—
LAN2	*LYN*	chr8:56922520:C>T	c.C1327T	p.R443C	13	34	—	—	—
NBLS	*ITK*	chr5:156672982:A>G	c.A1606G	p.S536G	15	27.5	—	—	—
SBJ12	*ROR1*	chr1:64643504:C>A	c.C1780A	p.H594N	9	24.5	—	—	—
SBJ12	*TYK2*	chr19:10467271:G>A	c.C2590T	p.R864C	18	33	—	—	—
SKNAS	*FLT3*	chr13:28599073:C>G	c.G2215C	p.G739R	18	24	—	—	—
SKNBE	*FES*	chr15:91437168:C>T	c.C2032T	p.R678C	16	28.7	0.0001	—	—
SKNSH	*ALK*	chr2:29443695:G>T	c.C3522A	p.F1174L	23	25.3	—	COSM28055	—
STANB1	*FGFR1*	chr8:38274849:G>T	c.C1371A	p.N457K	11	29.7	—	COSM1284968	—
NB3	ALK*	chr2:29432664:C>T	c.G3824A	p.R1275Q	25	35	—	COSM28056	—

Abbreviations: ALK*, mutation reported in dbSNP with rs113994087; CADD, Combined Annotation Dependent Depletion (v1.3); Cosmic, Catalogue of Somatic Mutations in Cancer (v70); Max Population Frequency, Maximum allele frequency in 1000 Genomes, ESP6500, ExAC, CG46; NB, Neuroblastoma; Variant Allele Frequency, Frequency of mutant allele in tumour tissue.

*P*‐value: Fisher Exact Test *P*‐value of somatic variants called by VarScan.

**FIGURE 1:**
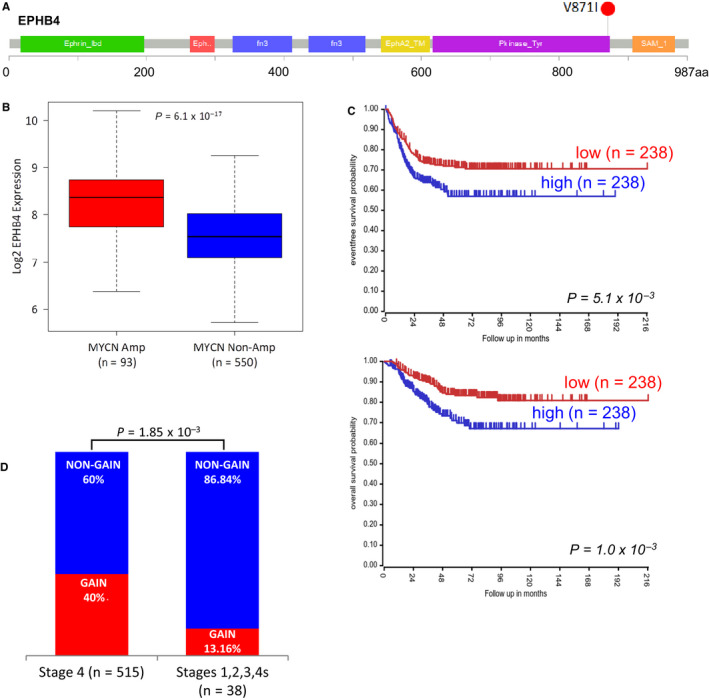
Expression, survival rate and copy number alterations of *EPHB4* gene in public data sets. A, Schematic map of EPHB4 protein domains and localization of V871I somatic mutation analysed in this study. B, Log2 transformed *EPHB4* gene expression levels in the GSE45547 data set. Samples were grouped according to the *MYCN* amplification status. C, Event‐free and overall survival rates in GSE45547 samples stratified based on *EPHB4* median expression value. D, Box plot showing Log Ratio values found in GSE103123. Grey dashed line at Log Ratio = 0.2 represents the copy number gain cut‐off used for CGH arrays whereas grey dotted line at Log Ratio = 0.15 shows the cut‐off used for copy number gain calls in SNP arrays

Interestingly, we found two mutations in *EPHB4* (V871I) and in *EphB6* (A417S) genes, both involved in axon guidance pathway. The variant V871I in the kinase domain of EPHB4 showed a high pathogenic score (Figure 1A and Table 1). The same tumour analysed by whole‐exome sequencing in our previous study^11^ confirmed the variant V871I.

The screening of kinome regions in NB cell lines resulted in 11 filtered mutations in eight genes. Here, we detected four additional *ALK* mutations. Of these, three were F1174L changes, and the other was the R1275Q change (Table 1). We also found a mutation (N457K) in *FGRF1* gene already found in other studies.^5^, ^11^, ^35^

An analysis of public NB gene expression data set (GEO Id: GSE45547)^36^ demonstrated that *EPHB4* expression positively correlated with *MYCN* amplification. Indeed, we found higher expression in *MYCN* amplified NBs compared with *MYCN* non‐amplified samples (*P* = 6.1 × 10^−17^) (Figure 1B). Survival analysis showed that high *EPHB4* expression reduced both overall and event‐free survival probabilities of NB patients (*P* = 1.0 × 10^−3^ and *P* = 5.1 × 10^−3^, respectively) (Figure 1C). To evaluate the independence of *EPHB4* gene expression, and its effect on patient survival, from the *MYCN* amplification status, we restricted the survival analysis to patients without *MYCN* amplification. We confirmed the trend of association between high *EPHB4* expression and reduced overall and event‐free survival rates but with non‐statistically significant values (*P* = 1.01 × 10^−1^ and 6.2 × 10^−2^, respectively, Figure S1A,B). Moreover, we surveyed the GSE3446 data set of 102 non‐*MYCN* amplified NBs published,^37^ and we again observed that high *EPHB4* expression reduced event‐free survival probabilities of NB patients (*P* = 6.5 × 10^−4^; Figure S2). Moreover, we evaluated the presence of copy number variants involving *EPHB4* region in a public data set of CGH and SNP arrays from a previous study (GEO Id: GSE103123).^31^ We found that the 40% (206/515) of stage 4 NBs showed a copy number gain whereas only the 13.16% of the lower stages NBs (5/33) had a copy number gain event (*P* = 1.85 × 10^−3^; Chi‐square test) (Figure 1D).

### 3.2 Expression analysis of EPHB4 in NB cell lines

**FIGURE 2:**
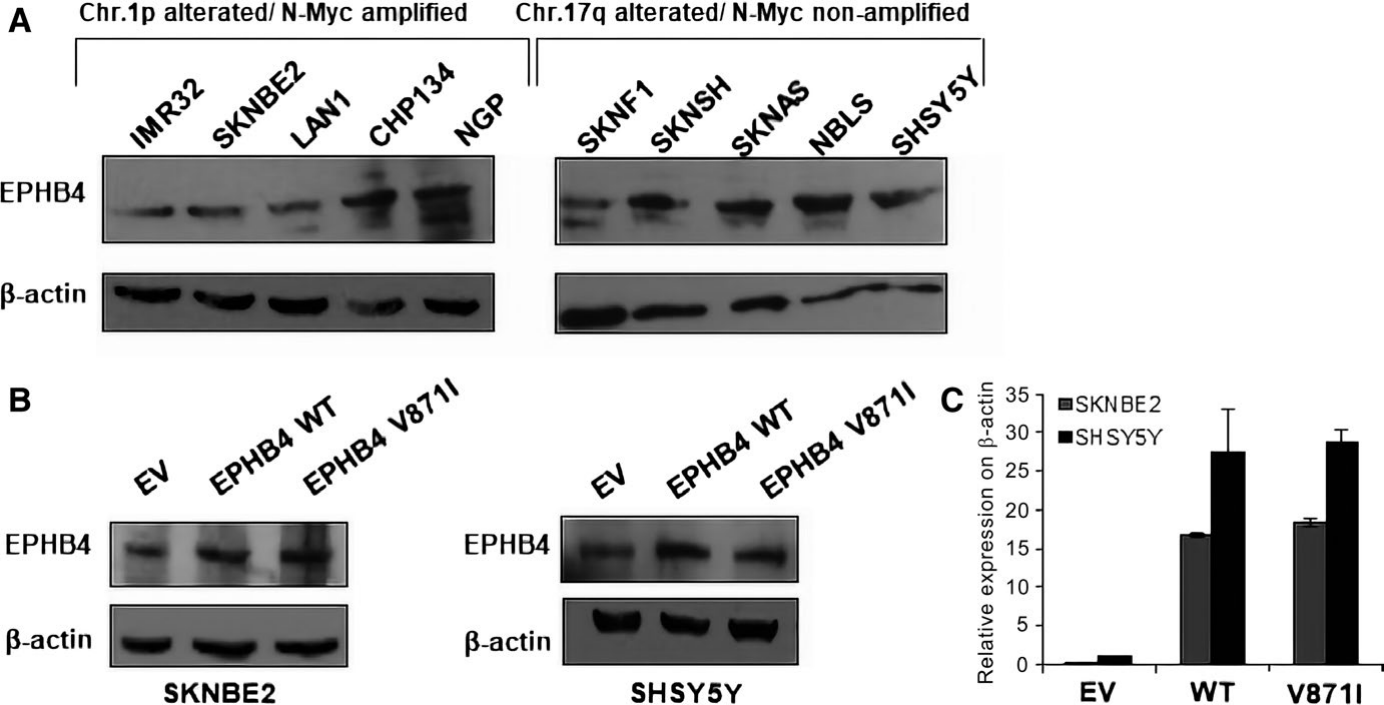
*EPHB4* expression in NB cell lines. A, Representative Western blotting of EPHB4 in several NB cell lines divided into Chr.1p imbalance/*MYCN* amplification and Chr.17q imbalance/no *MYCN* amplification. The β‐actin expression is shown for loading normalization. B, Representative Western blotting of EPHB4 protein in EPHB4‐EV, EPHB4‐WT and EPHB4‐V871I stable clones in SKNBE2 and SHSY5Y NB cell lines. The β‐actin expression is shown for loading normalization. C, Quantification of *EPHB4* mRNA levels normalized to *β‐actin* in EPHB4‐EV, EPHB4‐WT and EPHB4‐V871I stable clones in SKNBE2 and SHSY5Y NB cell lines. Data are presented as a mean ± SD. EV, empty vectorNB, Neuroblastoma

In order to select the cell lines to perform the functional characterization of EPHB4‐V871I variant, we firstly analysed the expression of EPHB4 in several NB cell lines. We divided our cell lines based on *MYCN* amplified and Chr1p alterations (IMR32, SKNBE2, LAN1, CHP134 and NGP) and *MYCN* non‐amplified and Chr17q alterations (SKNF1, SKNSH, SKNAS, NBLS, SHSY5Y) (Figure 2A). We selected SKNBE2 in the first group and SHSY5Y in the second one. To evaluate the expression of the EPHB4 mutant, we cloned EPHB4 wild‐type (WT) and EPHB4 mutant V871I (MUT) in pCMV6‐tag1. We then modelled our patient's genotype in vitro by stable clone transfection of WT and mutant EPHB4 expression plasmids into SKNBE2 and SHSY5Y cells. We found that the mutation did not impair *EPHB4* expression at the mRNA and protein level (Figure 2B,C).

### 3.3 EPHB4‐V871I affects proliferation and migration of NB cell lines

**FIGURE 3:**
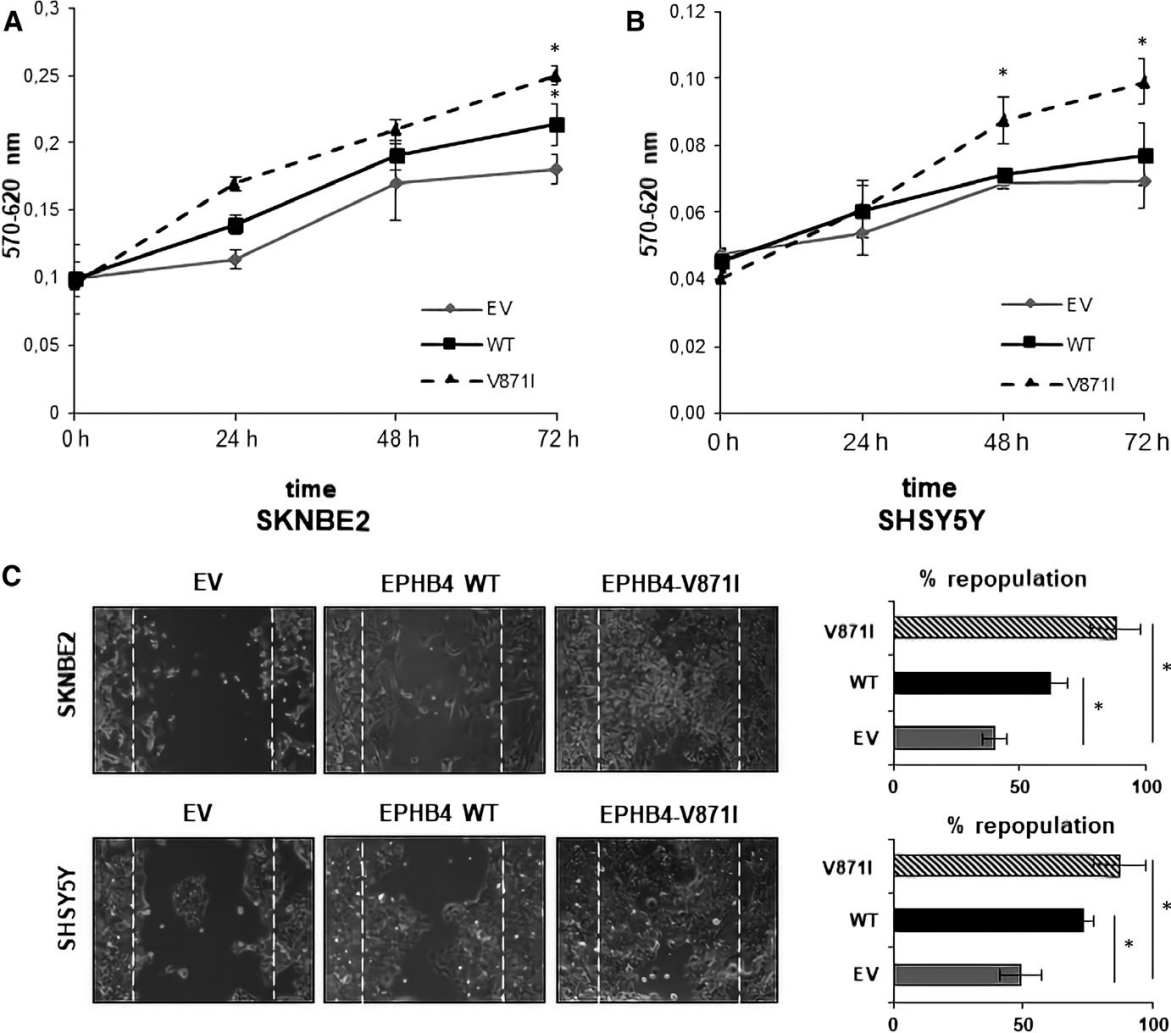
Proliferation and migration analysis of EPHB4‐V871I in NB cells. A, Proliferation assays by MTT in SKNBE2 cell line showing empty vector stable clone (EV) in grey line, EPHB4‐WT stable clone in black line and EPHB4‐V871I stable clone in dotted line. The data shown are mean ± SD from three independent experiments, each carried out in sixfold. * *P*‐value < .05 EPHB4‐V871I vs EPHB4‐WT, EPHB4‐V871I vs EV, EPHB4‐WT vs EV at 72 h. B, Proliferation assays by MTT in SHSY5Y cell line showing empty vector stable clone (EV) in grey line, EPHB4‐WT stable clone in black line and EPHB4‐V871I stable clone in dotted line. The data shown are mean from three independent experiments, each carried out in sixfold. * *P*‐value < .05 EPHB4‐V871I vs EPHB4‐WT at 48 h and 72h. * *P*‐value < .05 EPHB4‐V871I vs EV at 48 and 72 h. C, Representative images of monolayer wound healing of empty vector stable clone (EV), EPHB4‐WT stable clone and EPHB4‐V871I stable clone in SHSY5Y and SKNBE2 cells after 48 hours. * *P*‐value < .05 EPHB4‐V871I vs EPHB4‐WT, EPHB4‐WT vs EV in both SKNBE2 and SHSY5Y NB cell lines. Magnification, 100×. EV, empty vectorNB, Neuroblastoma

Due to EPHB4 involvement in tumour angiogenesis, growth and metastasis,^21^ we speculated on its potential regulation of cellular proliferation, cell migration and anchorage‐independent growth in vitro. The proliferation assay by MTT showed increased propagation rate of SKNBE2 cells stably overexpressing EPHB4‐WT compared with EV. Interestingly, cells stably overexpressing EPHB4‐V871I exhibited increased proliferation rate compared with both EV and EPHB4‐WT at 72 hours in (Figure 3A). The same results were confirmed at 48 and 72 hours in the SHSY5Y cell lines (Figure 3B).

The migration potential was firstly assayed by wound‐healing experiments demonstrating increased migration properties of EPHB4‐V871I compared with both EV and EPHB4‐WT in both cell lines (Figure 3C). Indeed, the percentage of cells repopulation is higher in EPHB4‐V871I compared with both EPHB4‐WT and EV.

**FIGURE 4:**
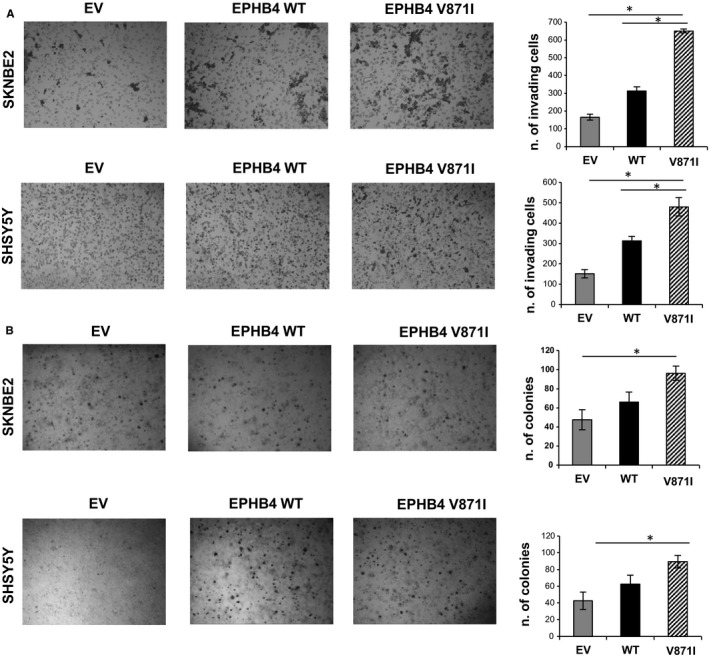
Two‐dimensional migration properties and anchorage‐independent growth analysis of EPHB4‐V871I in NB cells. A, Representative images of empty vector stable clone (EV), EPHB4‐WT stable clone and EPHB4‐V871I stable clone in SHSY5Y and SKNBE2 cells for migration through a porous membrane toward foetal bovine serum‐containing medium (left panel), with relevant quantification (right panel). Magnification, 100×. * *P*‐value < .05 EPHB4‐V871I vs EPHB4‐WT, EPHB4‐WT vs EV, EPHB4‐V871I vs EV. Data are means + standard errors of three independent experiments. B, (left panel) Representative images of empty vector stable clone (EV), EPHB4‐WT stable clone and EPHB4‐V871I stable clone in SHSY5Y and SKNBE2 cells for colony formation in soft agar assays with the plot (right panel) showing the colonies counted as means ± SD from three independent experiments. * *P*‐value < .05 EPHB4‐V871I vs EPHB4‐WT, EPHB4‐V871I vs EV. EV, empty vector; NB, Neuroblastoma

We further analysed the migration properties of EPHB4 mutant clone by two‐dimensional migration experiments. EPHB4‐V871I showed an increased number of migrating cells compared with EV and EPHB4‐WT in both cell lines (Figure 4A).

We further analysed the anchorage‐independent growth of EPHB4‐MUT by colony formation assay in soft agar. EPHB4‐V871I showed an increased number of colonies compared with WT and EV in both cell lines (Figure 4B).

### 3.4 EPHB4‐V871I increases the expression of some target genes and enhance the phosphorylation of ERK1‐2 pathway

**FIGURE 5:**
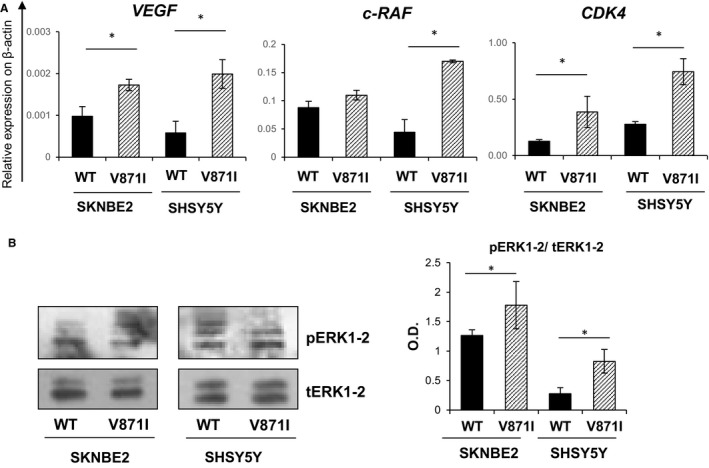
Analysis of target genes and ERK pathway of EPHB4‐V871I in NB cells. A, Quantification of *VEGF, c‐RAF and CDK4* mRNA level in EPHB4‐WT and EPHB4‐V871I stable clone in SHSY5Y and SKNBE2 cell lines. * *P*‐value < .05 EPHB4‐V871I vs EPHB4‐WT. Data are means ± standard deviation. B, Representative immunoblots are showing pERK1/2 and tERK1/2 protein in EPHB4‐WT and EPHB4‐V871I stable clone in SHSY5Y and SKNBE2 cell lines (right panel). Quantification by densitometric analysis of three separate Western blots with similar results (left panel). Data are means ± standard deviation. *P < .05, EPHB4‐V871I vs EPHB4‐WT. NB, Neuroblastoma

We then tried to study possible targets of EPHB4. We studied three EPHB4 downstream target genes by analysing the mRNA levels of: *VEGF, c‐RAF* and *CDK4* genes by qRt‐PCR. All three of these genes showed significantly higher levels of expression for EPHB4‐V871I compared with EPHB4‐WT for both the cell systems, SKNBE and SHSY5Y (Figure 5A). We further assessed the phosphorylation of ERK1‐2 pathway by Western blotting and we found increased phosphorylation status in EPHB4‐V871I compared with EPHB4‐WT for both cell systems, SKNBE and SHSY5Y (Figure 5B).

### 3.5 Treatment with TK inhibitors rescues the phenotype induced by EPHB4‐V871I in NB cell lines

**FIGURE 6:**
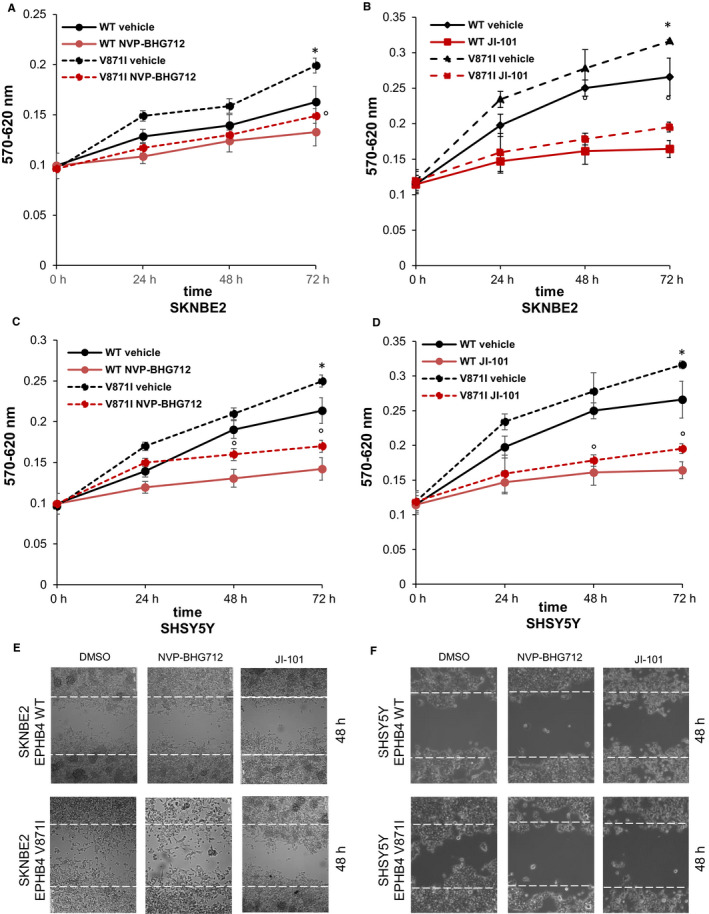
Rescue of the phenotype by treatment with EPHB4 tyrosine kinases inhibitors, NVP‐BHG712 and JI‐101, of EPHB4‐V871I in NB cells. A, Proliferation assays by MTT in SKNBE2 cell line showing: EPHB4‐WT stable clone treated with the vehicle‐DMSO in black line; EPHB4‐WT stable clone treated with NVP‐BHG712 in red line; EPHB4‐V871I stable clone treated with the vehicle‐DMSO in black dotted line; EPHB4‐V871I stable clone treated with NVP‐BHG712 in red dotted line. The data shown are mean from three independent experiments, each carried out in sixfold. *P < .05, EPHB4‐V871I treated with vehicle‐DMSO vs EPHB4‐V871I treated with NVP‐BHG712, °*P* < .05 EPHB4‐WT treated with vehicle‐DMSO vs EPHB4‐WT treated with NVP‐BHG712. B, Proliferation assays by MTT in SKNBE2 cell line showing: EPHB4‐WT stable clone treated with the vehicle‐DMSO in black line; EPHB4‐WT stable clone treated with JI‐101 in red line; EPHB4‐V871I stable clone treated with the vehicle‐DMSO in black dotted line; EPHB4‐V871I stable clone treated with JI‐101 in red dotted line. The data shown are mean from three independent experiments, each carried out in sixfold. *P < .05, EPHB4‐V871I treated with vehicle‐DMSO vs EPHB4‐V871I treated with JI‐101, °*P* < .05 EPHB4‐WT treated with vehicle‐DMSO vs EPHB4‐WT treated with JI‐101. C, Proliferation assays by MTT in SHSY5Y cell line showing: EPHB4‐WT stable clone treated with the vehicle‐DMSO in black line; EPHB4‐WT stable clone treated with NVP‐BHG712 in red line; EPHB4‐V871I stable clone treated with the vehicle‐DMSO in black dotted line; EPHB4‐V871I stable clone treated with NVP‐BHG712 in red dotted line. The data shown are mean from three independent experiments, each carried out in sixfold. *P < .05, EPHB4‐V871I treated with vehicle‐DMSO vs EPHB4‐V871I treated with NVP‐BHG712, °*P* < .05 EPHB4‐WT treated with vehicle‐DMSO vs EPHB4‐WT treated with NVP‐BHG712. D, Proliferation assays by MTT in SHSY5Y cell line showing: EPHB4‐WT stable clone treated with the vehicle‐DMSO in black line; EPHB4‐WT stable clone treated with JI‐101 in red line; EPHB4‐V871I stable clone treated with the vehicle‐DMSO in black dotted line; EPHB4‐V871I stable clone treated with JI‐101 in red dotted line. The data shown are mean from three independent experiments, each carried out in sixfold. *P < .05, EPHB4‐V871I treated with vehicle‐DMSO vs EPHB4‐V871I treated with JI‐101, °*P* < .05 EPHB4‐WT treated with vehicle‐DMSO vs EPHB4‐WT treated with JI‐101. E, Representative images of monolayer wound healing of EPHB4‐WT stable clone and EPHB4‐MUT stable clone in SKNBE2 cells after 48 hours of treatment with NVP‐BHG712 and JI‐101. Magnification, 100×. F, Representative images of monolayer wound healing of EPHB4‐WT stable clone and EPHB4‐V871I stable clone in SHSY5Y cells after 48 hours of treatment with NVP‐BHG712 and JI‐101. Magnification, 100×. NB, Neuroblastoma

To test if EPHB4 could be a promising druggable target for high‐risk NB, we treated our EPHB4 stable clones with two EPHB4 inhibitors, NVP‐BHG712 and JI‐101. We tested several concentrations for both drugs. We selected 0.5 µM of NVP‐BHG712 and 10 µM of JI‐101 in SKNBE2 cells and 1 µM of NVP‐BHG712 and 20 µM of JI‐101 for SHSY5Y cells because of no toxic effects. We firstly analysed the proliferation rate, and we found that the treatment with 0.5 µM of NVP‐BHG712 decreased the proliferation rate of EPHB4‐V871I compared with vehicle (DMSO) at 72 hours (Figure 6A). The treatment of EPHB4‐WT cells with NVP‐BHG712 also showed a decreased proliferation rate compared to vehicle (DMSO) in SHSY5Y at 72 hours (Figure 6A). The treatment with 10 µM of JI‐101 also decreased the proliferation rate of EPHB4 mutant clone compared to vehicle (DMSO) in SHSY5Y cells, and also of EPHB4‐WT compared to vehicle (Figure 6B) at 72 hours. The treatment of SKNBE2 clones with NVP‐BHG712 and JI‐101 showed similar effects by reducing the proliferation rate of EPHB4‐V871I compared with vehicle at 72 hours (Figure 6C,D).

Moreover, we assayed the migration potential after NVP‐BHG712 and JI‐101 treatment by wound‐healing experiments. We demonstrated that the treatment with the two inhibitors rescued the increased potential migration properties of EPHB4 mutant clone in both the cell lines. (Figure 6E,F).

## 4 DISCUSSION

We herein reported a novel somatic non‐synonymous variant, V871I, in the *EPHB4* gene occurring in our high‐risk NB cohort. The variant is associated with putative structural alterations and effects on proliferation and migration properties of NB cells.

*EPHB4* gene codified for the Ephrin type‐B receptor four which binds to transmembrane ephrin‐B family ligands residing on adjacent cells.^38^ It belongs to the EphB subfamily, that is the largest of receptor tyrosine kinases, comprises of six members (EphB1 to 6).^22^ To note, in a previous study on kinome expression profiling of NB tumours, it was observed a marked overexpression of *EPHB2* gene in a subset of ultra‐high‐risk NB, with highly aggressive clinical behaviour that not adequately respond to standard treatments.^20^ Similarly, *EPHB4* overexpression characterized several tumours such as breast, prostate, colon, uterus, melanoma and ovarian one.^23^, ^38^, ^39^, ^40^ EPHB4 plays a crucial role in cardiovascular development and regulates vascularization in cancer‐promoting angiogenesis, tumour growth and metastasis.^41^ Several *EPHB4* mutations have previously been identified in solid and haematological tumour specimens.^42^, ^43^, ^44^ Many other variants in other types of tumours and cell lines have been identified and catalogued in “The Cancer Genome Atlas project”.^24^ EPHB4 has been associated with tumour angiogenesis, growth and metastasis, thus making it a valuable and attractive target for drug design for therapeutic applications.^21^ However, EPHB4 mutations were not described until now in NB.

Our custom kinome targeted sequencing on a total of 45 NB normal‐primary tumour matched pairs, and 9 NB cell lines identified 11 filtered mutations in eight genes. According to previous sequencing screenings,^11^ we observed very few somatic mutations in coding regions. Our data further confirmed ALK as most frequently mutated kinase gene in NB. Furthermore, here we report the *FGFR1* mutation N457K, already found in our previous work and other sequencing studies on primary and relapsed NBs.^5^, ^11^ Despite *FGFR1* mutation remains infrequent in NB, we can speculate on the presence of a mutational hot‐spot in the kinase domain of *FGFR1. FGFR1* mutations could be functional not only for the development but also for the selection of resistant and metastatic clones in NB.

Interestingly, we found two variants in *EPHB4* (V871I) and *EPHB6* (A417S), both genes are involved in axon guidance pathway. These findings suggest that defects in genes involved in neuronal growth are an important category of tumour‐driving events in NB. Of note, we already reported another *EPHB4* somatic pathogenic mutation (P257L) in our previous study comprising a total of 82 NB samples.^11^

*EPHB4* expression positively correlated with *MYCN* amplification (a marker of high‐risk and high‐stages NB). Indeed, we found higher *EPHB4* expression in *MYCN* amplified NBs compared with *MYCN* non‐amplified samples. Survival analysis showed that high *EPHB4* expression reduced both overall and event‐free survival probabilities of NB patients. Moreover, we evaluated the presence of copy number variants involving *EPHB4* region in a public data set of CGH and SNP arrays from the previous study.^30^ We found that 40% of stage 4 NBs showed a copy number gain, whereas only the 13.16% of the lower stages NBs had a copy number gain event. These data suggest that diverse genetic alterations such as point mutations and genomic gains can act on the tumorigenic potential of the *EPHB4* gene.

Overall, this data suggested a potential role of *EPHB4* in NB progression. Subsequently, we characterized the EPHB4‐V871I variant in respect with EPHB4‐WT in two NB cell lines, SHSY5Y and SKNBE. The two cell lines were selected for the different levels of endogenous *EPHB4* expression (higher in SHSY5Y than in SKNBE2) and different genetic background (SKNBE2 carried *MYCN* amplification while SHSY5Y are negative for *MYCN* amplification). Our data demonstrated that EPHB4‐V871I enhances the proliferation rate of both NB cell lines suggesting a gain of function of the mutant. Moreover, the migration potential, assessed by both wound‐healing and two‐dimensional migration tests, was increased in EPHB4‐V871I in respect with EPHB4‐WT in both cell lines. This data confirmed the in vivo data that correlated the gain of *EPHB4* with stage 4 and high *EPHB4* expression to decreased survival rate in NB patients. Finally, we also tested the anchorage‐independent growth of our cell systems, demonstrating that EPHB4‐V871I can enhance the colony formation potential of both NB cell lines. Anchorage‐independent growth is one of the hallmarks of malignant cell transformation,^45^ so this data confirmed the increased malignant potential of EPHB4‐V871I mutant. We speculated that the variant V871I, localized in the tyrosine kinase domain, could alter the three‐dimensional structure of the domain, enhancing the downstream signalling pathways. For this reason, we analysed three target genes of EPHB4: *VEGF*, c‐*RAF* and *CDK4*. It has been demonstrated that EPHB4 regulates cell proliferation mediated by the Abl‐CyclinD1 pathway in neural stem cells by increasing CDK4 expression.^46^ Moreover, EPHB4 regulates angiogenesis by VEGF and converging on its downstream signalling and tuning the phosphorylation of ERK1/2.^47^ The MAPK signalling pathway is composed of the downstream signalling molecules RAF, MEK and extracellular signal‐regulated kinase (ERK).^48^ We found that the variant V871I enhances the expression of the mRNA levels of all the three genes in both the cell lines analysed. To confirm this, the phosphorylation of ERK1‐2 was also increased in EPHB4 mutant compared to WT. This data demonstrated that downstream signalling pathway of EPHB4‐V871I is enhanced compared with EPHB4‐WT.

Identifying genomic and genetic variants in Eph receptors’ gene family may have valuable clinical implications since they are attractive targets for therapeutic applications in cancer.^49^ Many strategies have been applied to evaluate the interference of tumour‐promoting effects or the enhancement of tumour suppressive effects. The inhibition of the Eph‐ephrin system may be particularly useful for anti‐angiogenic therapies.^50^ The ephrin‐binding pocket in the extracellular N‐terminal domain of Eph receptors and the ATP‐binding pocket in the intracellular kinase domain could be potential binding sites for peptides and small molecules.^51^ Consequently, multi‐targeted tyrosine kinase inhibitors could also inhibit the kinase activity of EPHB4; some of these, such as EXEL‐7647 and dasatinib, are in clinical trials.^50^

Additionally, some new EPHB4 inhibitors have been obtained from kinase inhibitor libraries. NVP‐BHG712 was reported to target EPHB4 and to also inhibit VEGF in vivo.^51^ NVP‐BHG712 also shows a synergistic effect with other chemotherapy drugs for solid tumours. JI‐101 is an oral multi‐kinase inhibitor that was demonstrated to inhibit VEGFR2, platelet‐derived growth factor receptor β (PDGFR‐β), and EPHB4.^52^ It was used in a phase I trial in patients with advanced solid tumours.^52^ We used these two drugs to verify the possible rescue of the phenotype observed in EPHB4‐V871I mutant. We assessed the proliferation rate of EPHB4‐V871I after treatment with both drugs showing restoring of the proliferation rate at basal levels in both the cell lines. The rescue of the phenotype was also obtained regarding the migration properties after NVP‐BHG712 and JI‐101 treatment. Thus, the inhibition of EPHB4 tyrosine kinase activity rescued the malignant phenotype of EPHB4‐V871I. So, we speculated on the possible use of these drugs in future therapeutic application to treat those patients carrying *EPHB4* gain‐of‐function somatic mutations.

We demonstrated that our t‐NGS panel comprising the kinase domains of TKs could be a promising screening tool to identify druggable mutations in TKs in NB. Moreover, our data suggest that genomic/genetic alterations can promote NB tumour progression by the activation of the druggable gene *EPHB4*.

## CONFLICT OF INTERESTS

We have nothing to disclose.

## AUTHORS' CONTRIBUTIONS

IA and MC designed and conducted the study, and prepared the manuscript; VAL performed the NGS bioinformatic analysis; FM performed all the cell lines experiments, qRT‐PCR and Western blotting; BRE performed all the drugs treatments and correlated analysis; DF performed the cloning experiments; AI provided critical review of the manuscript.

## Supporting information

Fig S1

Fig S2

Table S1‐S4
