# Supplementary figures and images for: Kinome multigenic panel identified novel druggable EPHB4‐V871I somatic variant in high‐risk neuroblastoma
Source: J Cell Mol Med. 2020 Apr 26;24(11):6459–71. doi: 10.1111/jcmm.15297 (PMC7294133; doi:10.1111/jcmm.15297)

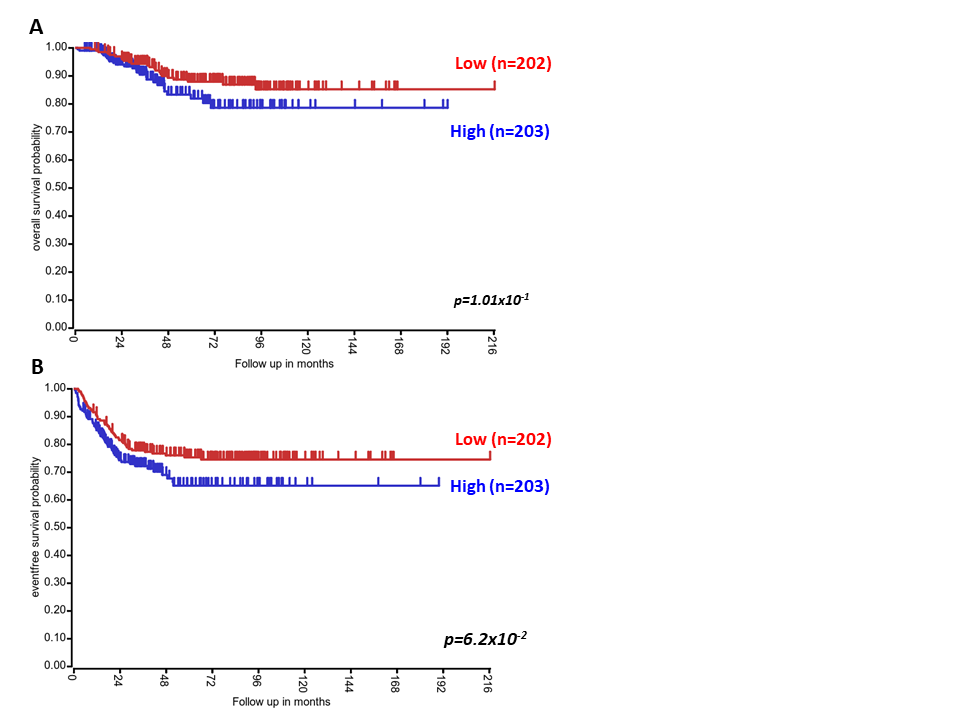

Supplement: Supplementary file 1 — Fig S1 [file JCMM-24-6459-s001.tif]

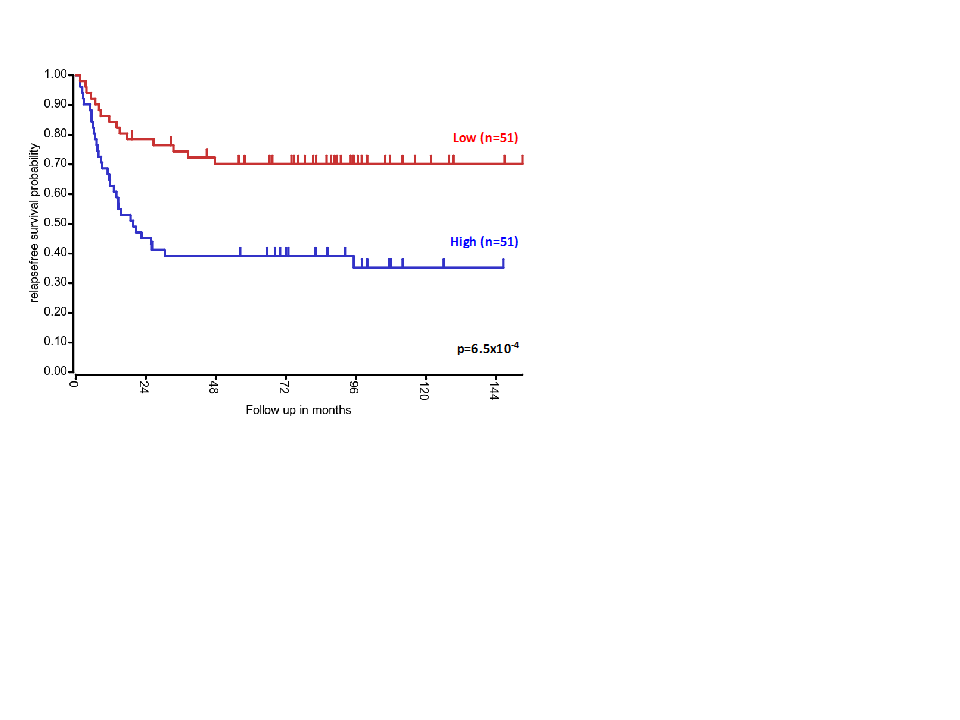

Supplement: Supplementary file 2 — Fig S2 [file JCMM-24-6459-s002.tif]
