# Supplementary material for: Kinome multigenic panel identified novel druggable EPHB4‐V871I somatic variant in high‐risk neuroblastoma
Source: J Cell Mol Med. 2020 Apr 26;24(11):6459–71. doi: 10.1111/jcmm.15297 (PMC7294133; doi:10.1111/jcmm.15297)
Supplement: Supplementary file 4 [file JCMM-24-6459-s004.docx]

**Kinome multigenic panel identified novel druggable EPHB4-V871I somatic variant in high-risk neuroblastoma**

Immacolata Andolfo^1,2^, Vito Alessandro Lasorsa^1,2^, Francesco Manna^1,2^, Barbara Eleni Rosato^1,2^, Daniela Formicola^3^, Achille Iolascon^1,2^, Mario Capasso^1,2,3^.

1 Department of Molecular Medicine and Medical Biotechnologies, University of Naples Federico II, Naples, Italy

2 CEINGE, Biotecnologie Avanzate, Naples, Italy

3 IRCCS SDN, Napoli

**Supplementary material contains:**

- **Figure S1**
- **Figure S2**
- **Table S1**
- **Table S2**
- **Table S3**
- **Table S4**
- **Supplementary legends**

**Supplementary Figure 1. *EphB4* dependent survival probabilities in non-MYCN amplified samples of GSE45547** **public data set.**

**A.** Overall survival rate in 405 non-*MYCN* amplified samples stratified on the basis of *EphB4* median expression value. **B.** Event-free survival rate in 405 non-*MYCN* amplified samples stratified on the basis of *EphB4* median expression value.

**Supplementary Figure 2. *EphB4* gene expression and survival probabilities in GSE3446 public data set.**

Relapse-free survival rates in 102 samples stratified on the basis of *EphB4* median expression value.
